# Antibacterial Applications of Nanomaterials

**DOI:** 10.3390/nano13091530

**Published:** 2023-05-02

**Authors:** Krasimir Vasilev

**Affiliations:** College of Medicine and Public Health, Flinders University, Bedford Park, SA 5042, Australia; krasimir.vasilev@flinders.edu.au; Tel.: +61-424597759

In the 21st century, infections remain a major problem for society and are one of the leading causes of mortality. Beyond healthcare, undesired bacterial contamination, attachment, and colonization cause significant issues in many other areas of everyday life and industry. Examples include the food manufacturing and distribution and the marine industry. Despite the wide application of antibiotics, cleaning, and prophylactic practices, to this date, the problems with undesired bacterial adhesion have not found a complete solution. Moreover, the growing prevalence of antibiotic-resistant microorganisms threatens to escalate the problem over the coming years substantially.

The extent of the problem of infections and undesired bacterial contamination has triggered massive research efforts by researchers around the World in an attempt to find a solution. One area that holds substantial promise is nanotechnology. Over the last three decades, scientists and engineers have made exciting discoveries of natural and engineered nanoscale materials capable of eliminating bacteria or preventing their attachment to surfaces [1,2].

This Special Issue aims to serve as an exciting collection of primary research articles on the recent progress in the synthesis, fabrication, and utilization of nanoscale materials for antibacterial applications.

In this Special Issue, we selected ten research articles from research leaders that offer interesting nanotechnology-based platforms that provide exciting future direction for the field.

I will begin the summary of accepted papers with a research study by Bright et al. [3] which deals with an intriguing technology that nature has inspired. First, observed on insect wings, these surfaces can kill bacteria upon contact with sharp surface nanostructure without the help of any chemicals or drugs. Since their discovery, these type of surface nanostructures have been translated to many synthetic materials, such as silicon and titanium [4,5]. In their article, Bright and co-workers investigated the role of etching duration and sodium and potassium cations used during alkaline heat treatment on the topographical, physical, and bactericidal properties of a medical-grade titanium alloy. The authors determined the optimal time of hydrothermal processing in KOH and NaOH that provides optimal antibacterial performance. The reported parameters can guide the biomedical industry and researchers in translating this nanoscale antibacterial surface modification to commercially used medical devices.

Several articles make use of the antibacterial properties of silver, which have been of massive interest over the last two decades [6]. Hurtuková et al. developed an antibacterial surface on flexible support by first sputtering a carbon base layer on PDMS, followed by sputtering silver. In the next step, the nanostructured surface was heat treated and activated by an excimer laser [7]. This process led to forming a surface with strong antibacterial properties against bacterial pathogens such as *S. epidermidis* and *E. coli*. In another interesting article, Beery et al. [8] developed silver nanoparticles incorporated in anion exchange resin beads. The authors generated beads with different silver loadings and showed that Ag-loaded resins made using 50 mM AgNO_3_ were able to eliminate 99% *E. coli* within three hours of exposure. The authors’ motivation for this work was to offer antibacterial resins that are easy to handle, have a long-term shelf life, are reusable, and reduce the risk of environmental contamination. Novel Bacitracin-Ag nanoclusters were prepared by Wang and co-authors [9] with the specific goal of targeting *Shigella flexneri*. Ag nanoclusters have recently become a hot research topic due to their high antibacterial potency [10,11]. Wang et al. found that the Bacitracin-Ag nanoclusters were very potent against this pathogen and determined that the minimum inhibitory concentration (MIC) and minimum bactericidal concentration (MBC) were 0.03 mg/mL and 4 mg/mL, respectively. The authors suggest that the nanoclusters can serve as a novel antimicrobial agent for application in the food industry. Moorthy et al. reported a facile and green synthesis method of ecologically viable silver nanoparticles using aqueous and ethanolic) dried bitter gourd (Momordica charantia) fruit extract [12]. The bitter gourd fruit extract was utilized as both reducing and capping agents. The authors claim that the synthesized silver nanoparticles possessed synergistic antioxidant and antibacterial action and had MIC of 4 µg/mL against drug-resistant bacterial strains. In another interesting paper, Murei and co-workers synthesized silver nanoparticles by chemical and biological methods and then conjugated them with Pyrenacantha grandiflora extracts [13]. The prepared silver nanoparticles had very potent antibacterial activity against multi-drug resistant pathogens. Very low MIC of 0.0063 mg/mL against MRSA was measured when biologically synthesized silver nanoparticles were conjugated with acetone and water extracts. The chemically synthesized silver nanoparticles had lowest MIC of 0.0063 mg/mL against *E. coli* when conjugated with acetone and methanol extracts.

David et al. decorated multi-walled carbon nanotubes with several different types of nanoparticles (NPs) to generate hybrid materials with improved antimicrobial activity [14]. The authors found that the presence of zinc oxide and silver nanoparticles enhanced the antimicrobial properties of the carbon nanotubes against clinically relevant microbial strains.

Yougbaré and co-workers synthesized visible-light-activated metallic molybdenum disulfide nanosheets and plasmonic gold nanorods [15]. The obtained material was denoted as MoS2@AuNRs. The photothermal and photodynamic activity of MoS2@AuNRs were studied against *E. coli*. Combining photothermal and photodynamic therapy using the MoS2@AuNR nanocomposites had higher antibacterial activity than photothermal or photodynamic therapy alone. The authors proposed that the light-activated MoS2@AuNR nanocomposites’ remarkable synergistic effect during the combination of photothermal and photodynamic therapy can provide an alternative approach to fight bacterial infections. 

Tan et al. fabricated plasmonic gold nanoisland film for bacterial theragnostic [16]. The films exhibited greater capture efficiency towards *E. coli* than unmodified glass substrate. Due to their nanostructured nature, the films could be used as a surface-enhanced Raman scattering (SERS) sensor to enhance the Raman signal of *E. coli*. Furthermore, the films displayed excellent capacity to kill *E. coli* photothermally, thus providing a promising theragnostic platform.

Lastly, Hui and co-workers incorporated five plant essential oils on ZnO/palygorskite nanoparticles by a simple adsorption process to form organic–inorganic nanocomposites with antibacterial properties [17]. The carvacrol/ZnO/palygorskite was the most efficient of the nanocomposites with minimum inhibitory concentration of 0.5 mg/mL and 1.5 mg/mL against *E. coli* and *S. aureus*, respectively.

As a final note, the Editorial team would like to thank all contributing authors for providing such interesting articles for this Special Issue and making it a great success.
